# Supplementary material for: Application of an Integrated Single-Cell and Three-Dimensional Spheroid Culture Platform for Investigating Drug Resistance Heterogeneity and Epithelial–Mesenchymal Transition (EMT) in Lung Cancer Subclones
Source: Int J Mol Sci. 2025 Feb 19;26(4):1766. doi: 10.3390/ijms26041766 (PMC11855057; doi:10.3390/ijms26041766)
Supplement: Supplementary file 1 [file ijms-26-01766-s001.zip › Figure S1.pdf]

Supplementary Figure S1. *Distinct Morphological and Growth Profiles Characterize Three Principal Cisplatin-Resistant Subclones*

Among the 17 cisplatin-resistant subclones, three principal subtypes—Holoclone, Meroclone, and Paraclone—were identified, each exhibiting distinct morphological and growth characteristics. Holoclone subclones typically grew as small, compact clusters composed of the smallest cells among the three subtypes and were notable for producing abundant mucus. In contrast, Meroclone subclones expanded in a sheet-like pattern, featuring polygonal, moderately sized cells with only minimal mucus secretion. Paraclone subclones displayed a distinctly dispersed growth pattern, consisting of elongated or irregularly shaped cells that were the largest observed and did not secrete mucus. Despite their overarching classification into these three categories, each individual subclone demonstrated unique growth patterns and subtle phenotypic variations.

By establishing a comprehensive biobank of drug-resistant subclones, we create a valuable resource that enables systematic exploration of intratumoral heterogeneity and the complexity of resistance mechanisms. Such a repository not only preserves the unique biological and molecular signatures of these subclones but also facilitates longitudinal, multi-dimensional analyses. This approach allows for parallel evaluations of various therapeutic strategies, advancing our understanding of how different resistant phenotypes emerge, adapt, and interact under selective pressures. Ultimately, this biobank serves as a pivotal tool, driving innovation in targeted therapy design, combination treatment regimens, and personalized medicine protocols aimed at overcoming drug resistance in clinical settings.

Clone No.	Growth pattern	Day 7	10 cm Dish
(1) 0328 – 1 – C1	Paraclone	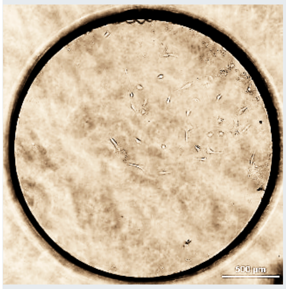	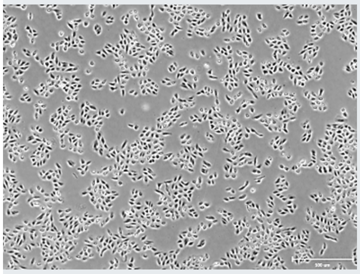
(2) 0328 – 1 – C4	Holoclone	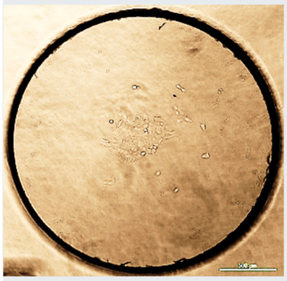	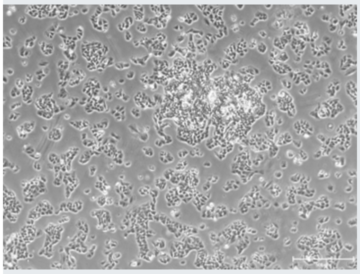
(3) 0328 – 1 – C6	Meroclone	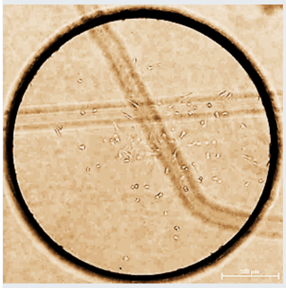	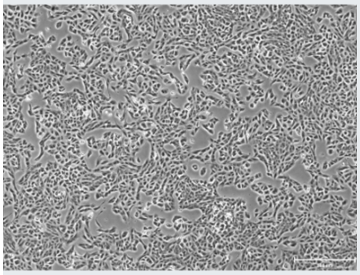

(4) 0328 – 1 – C12	Meroclone	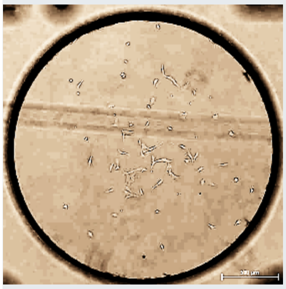	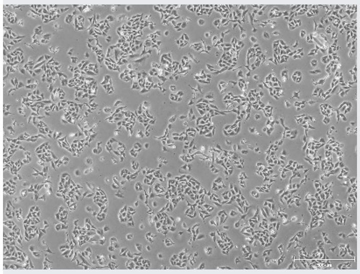
(5) 0328 – 1 – D12	Holoclone	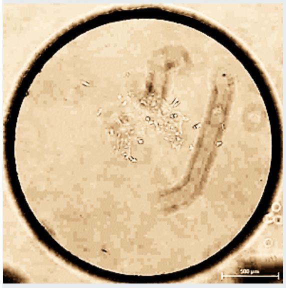	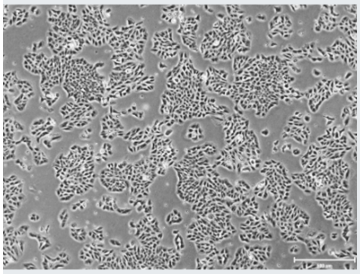
(6) 0328 – 2 – A12	Paraclone	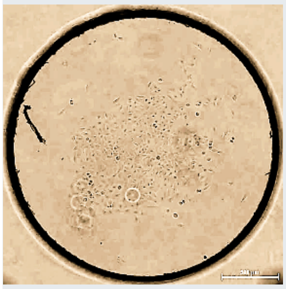	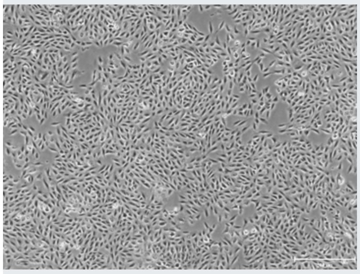
(7) 0328 – 2 – B14	Meroclone	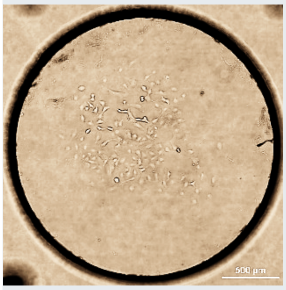	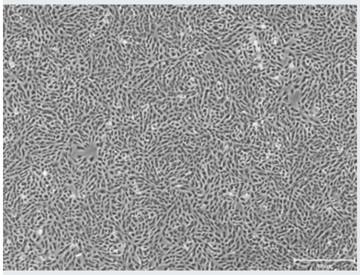
(8) 0328 – 2 – C1	Meroclone	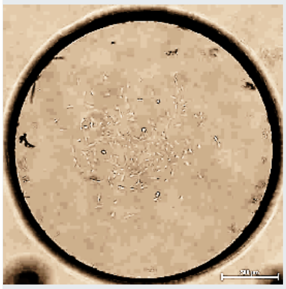	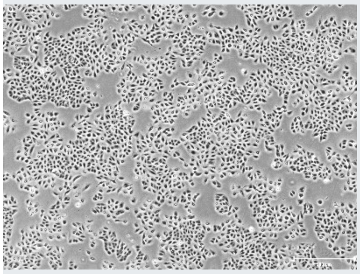

(9) 0328 – 2 – C15	Paraclone	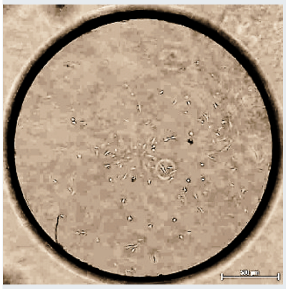	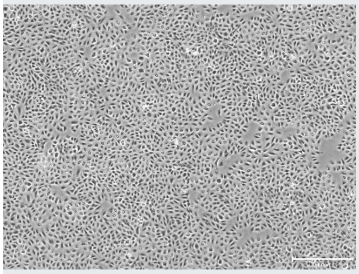
(10) 0328 – 3 – C11	Holoclone	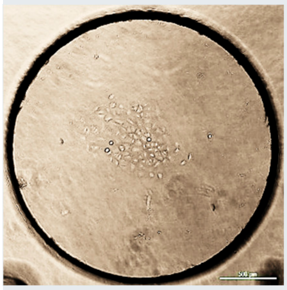	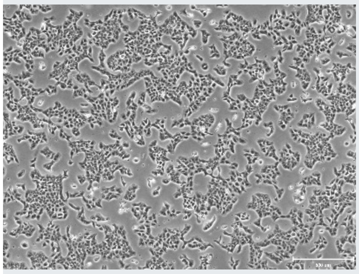
(11) 0328 – 3 – D15	Meroclone to Paraclone	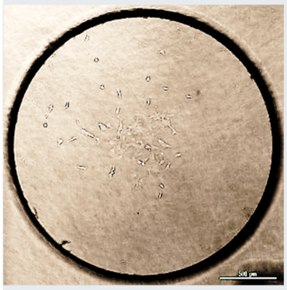	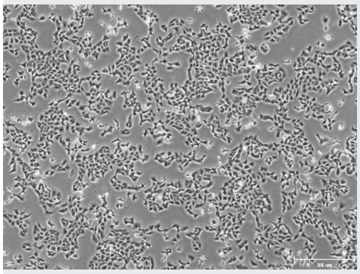
(12) 0406 – 1 – B13	Paraclone	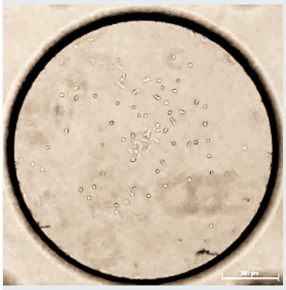	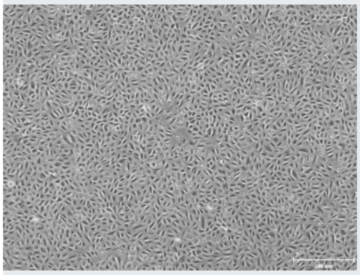
(13) 0406 – 2 – B14	Paraclone to Meroclone	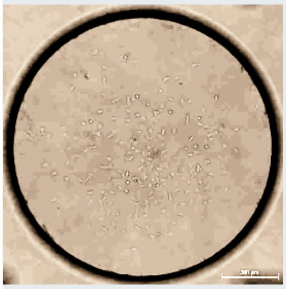	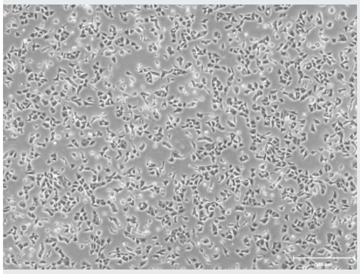

(14) 0406 – 2 – C8	Paraclone	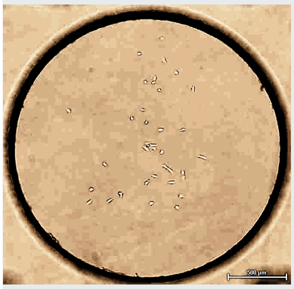	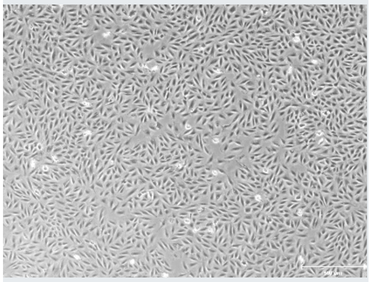
(15) 0328 – 2 – D4	Paraclone/	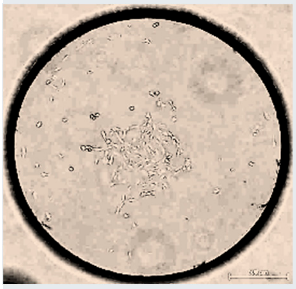	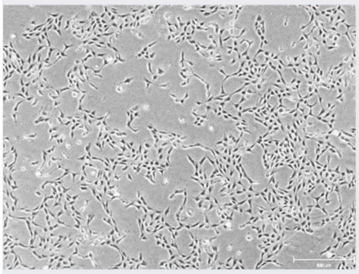
(16) 0406 – 4 – A12	Paraclone	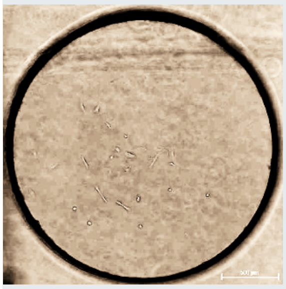	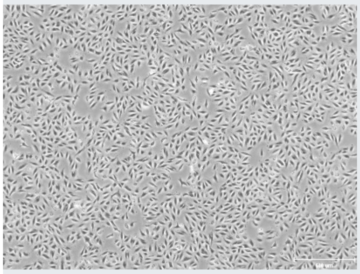
(17) 0406 – 4 – B2	Paraclone	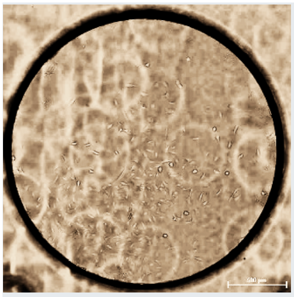	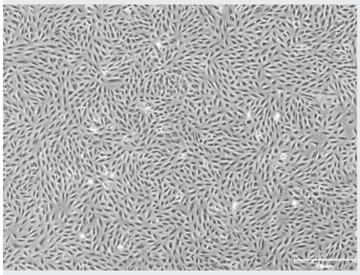
