# Supplementary material for: Application of an Integrated Single-Cell and Three-Dimensional Spheroid Culture Platform for Investigating Drug Resistance Heterogeneity and Epithelial–Mesenchymal Transition (EMT) in Lung Cancer Subclones
Source: Int J Mol Sci. 2025 Feb 19;26(4):1766. doi: 10.3390/ijms26041766 (PMC11855057; doi:10.3390/ijms26041766)
Supplement: Supplementary file 1 [file ijms-26-01766-s001.zip › Table S1-S3.pdf]

Table S1

Clinical Case Analysis: Genetic Variations and Targeted Therapy in Poorly Differentiated Lung Adenocarcinoma

In this study, we examined two patients diagnosed with poorly differentiated pulmonary adenocarcinoma, both presenting with malignant pleural effusions indicative of advanced disease and a poorer clinical prognosis. Genetic analyses revealed specific mutations potentially linked to aggressive tumor behavior and drug resistance. Based on these genetic profiles, the clinical team selected corresponding targeted therapies aiming to improve treatment efficacy and prolong survival.

These cases underscore the importance of genetic testing in lung cancer management. By identifying particular gene mutations, clinicians can tailor more precise treatment strategies and employ targeted agents more effectively, thereby increasing therapeutic efficacy and minimizing unnecessary adverse effects. Moreover, given the inherently high invasiveness and metastatic potential of poorly differentiated adenocarcinomas, the application of targeted therapies is especially critical. It offers a more focused approach to inhibit tumor growth and dissemination, ultimately enhancing patient outcomes.

	**Case 1**	**Case 2**
Clinical Stage	stage IVB, cT4N0M1c	stage IVA, cT4N3M1a
Pathological grading	Poorly differentiated pulmonary adenocarcinoma	Poorly differentiated pulmonary adenocarcinoma
NGS results	EGFR: Del 19	Exon 14 splice mutations
Drug application	Giotrif<br>IC50 of 60 nM	Capmatinib<br>IC50 of 6.5 nM

Table S2. Detailed List of Antibodies Used

Antibody name	clone	Dilute WB/Flow /IHC	Company
MDR-1	Rabbit pAb (GTX108370)	1:1000	Gene Tex, USA.
ABCG2	Rabbit pAb (GTX100437)	1:1000	Gene Tex, USA.
OCT4	Rabbit pAb (GTX101497)	1:500	Gene Tex, USA.
SOX2	Rabbit pAb (bs0523R)	1:500	Bioss Antibodies, USA.
PE-CD44	Mouse mab (338807)	1:100	BioLegend, USA.
PE-CD133	Mouse mab (393903)	1:50	BioLegend, USA.
E-cadherin	Rabbit pAb (GTX100443)	1:1000/1:300	Gene Tex, USA.
Vimentin	Rabbit pAb (GTX100619)	1:1000/1:500	Gene Tex, USA.
N-cadherin	Rabbit pAb (GTX127345)	1:1000/1:200	Gene Tex, USA.
Twist	Rabbit pAb (GTX127310)	1:500	Gene Tex, USA.
b-actin	Mouse mab (ab213262)	1:3000	Abcam, UK

Table S3. Detailed List of Primers Used

Oligo Title	Sequences (5′ to 3′)
GAPDH	qFP -AAGGTGAAGGTCGGAGTCAA
	qRP -AATGAAGGGGTCATTGATGG
MDR1	qFP -ATTCCTCGAGAAACTGCGAA
	qRP -TCACTTCAGGCAACCAG
Vimentin	qFP -CTTCAGAGAGAGGAAGCCGA
	qRP -ATTCCACTTTGCGTTCAAGG
E-cadherin	qFP -GCCGAGAGCTACACGTTCAC
	qRP -GTCGAGGGAAAAATAGGCTG
ABCG2	qFP -CTGAGATCCTGAGCCTTTGG
	qRP -TGCCCATCACAACATCATCT
CD133	qFP -CAGCAGAGAGCAGATGACCA
	qRP -TCCACAGAA ATTTACCTACAT TGG
SOX2	qFP -GGCAGCTACGCATGATGCAGGAGC
	qRP -CTGGTCATGGAGTTGTACTGCACG
Nanog	qFP -AATACCTCAGCCTCCAGCAGATG
	qRP -CTGCGTCACACCATTGCTATTCT
OCT-4	qFP -CGCACCACTGGCATTGTCAT
	qRP -TTCTCCTTGATGTCACGCAC
Snail-1	qFP -CTCTAGGCCCTGGCTGCTAC
	qRP -TCTGAGTGGGTCTGGAGGTG
